# Promoting electrochemical ammonia synthesis by synergized performances of Mo_2_C-Mo_2_N heterostructure

**DOI:** 10.3389/fchem.2023.1122150

**Published:** 2023-02-16

**Authors:** Tae-Yong An, Subramani Surendran, Sebastian Cyril Jesudass, Hyunjung Lee, Dae Jun Moon, Jung Kyu Kim, Uk Sim

**Affiliations:** ^1^ Hydrogen Energy Technology Laboratory, Korea Institute of Energy Technology (KENTECH), Naju, Republic of Korea; ^2^ Department of Science and Engineering, Chonnam National University, Gwangju, Republic of Korea; ^3^ School of Chemical Engineering, Sungkyunkwan University, Suwon, Republic of Korea; ^4^ Research Institute, NEEL Sciences, INC, Jeollanamdo, Republic of Korea; ^5^ Center for Energy Storage System, Chonnam National University, Gwangju, Republic of Korea

**Keywords:** ammonia electrosynthesis, electrocatalyst, electrochemical nitrogen reduction reaction, heterostructures, Mo_2_C catalyst, Mo_2_N catalyst

## Abstract

Hydrogen has become an indispensable aspect of sustainable energy resources due to depleting fossil fuels and increasing pollution. Since hydrogen storage and transport is a major hindrance to expanding its applicability, green ammonia produced by electrochemical method is sourced as an efficient hydrogen carrier. Several heterostructured electrocatalysts are designed to achieve significantly higher electrocatalytic nitrogen reduction (NRR) activity for electrochemical ammonia production. In this study, we controlled the nitrogen reduction performances of Mo_2_C-Mo_2_N heterostructure electrocatalyst prepared by a simple one pot synthesis method. The prepared Mo_2_C-Mo_2_N_0.92_ heterostructure nanocomposites show clear phase formation for Mo_2_C and Mo_2_N_0.92_, respectively. The prepared Mo_2_C-Mo_2_N_0.92_ electrocatalysts deliver a maximum ammonia yield of about 9.6 μg h^-1^ cm^-2^ and a Faradaic efficiency (FE) of about 10.15%. The study reveals the improved nitrogen reduction performances of Mo_2_C-Mo_2_N_0.92_ electrocatalysts due to the combined activity of the Mo_2_C and Mo_2_N_0.92_ phases. In addition, the ammonia production from Mo_2_C-Mo_2_N_0.92_ electrocatalysts is intended by the associative nitrogen reduction mechanism on Mo_2_C phase and by Mars-van-Krevelen mechanism on Mo_2_N_0.92_ phase, respectively. This study suggests the importance of precisely tuning the electrocatalyst by heterostructure strategy to substantially achieve higher nitrogen reduction electrocatalytic activity.

## 1 Introduction

The modern technical society holds a shear desire for cost-effective, eco-friendly, and reliable energy technologies to mitigate pollution and ensure energy sustainability (Fiksel et al., 2014). Researchers and technologists have conceded with the development of hydrogen energy which was abundantly available and displayed potential applications in various sectors (Yue et al., 2021). However, the storage and transport of valuable fuel became an exceptional threat which involved complex storage facilities and cryogenic systems (Zhou et al., 2005). Ammonia was then ascertained as the effective hydrogen carrier, which can hold up to 17.65% of its total mass (Avery et al., 1988; MacFarlane et al., 2020). Ammonia can be transported over long distances under ambient conditions and could also be utilized as fuels directly (Zhao et al., 2019). Nevertheless, ammonia is produced by the high energy-consuming Haber-Bosch (HB) process, which involves nitrogen and natural gas (CH_4_), resulting in greenhouse gas (GHG) evolution (Kandemir et al., 2013; Safari et al., 2019). Technologies for eco-friendly and sustainable ammonia production are yearned to achieve supremacy over green energy.

Electrochemical nitrogen reduction reaction (NRR) is the most viable and simple technology developed for ammonia production. NRR involves water, N_2_, and renewable electricity to generate ammonia over six proton-coupled electron transfer (PCET) steps (MacFarlane et al., 2020). NRR is facilitated over an electrocatalyst which must exhibit better activity, selectivity, and stability over NRR electrolysis (Cui et al., 2018). Since the triple bond of dinitrogen (N≡N) has a high dissociation energy of nearly 941 kJ mol^-1^, NRR requires large overpotentials, which are much lower than required for hydrogen evolution reaction (HER) (Ren et al., 2021). Hence, HER becomes an active competitor for NRR, resulting in a lower ammonia yield rate and Faradaic efficiency (FE). Various electrocatalyst designs were engineered to increase activity for NRR and selectivity towards ammonia without the intermediate formation and HER (Du et al., 2021).

Mo-based electrocatalysts have been analyzed as the effective NRR electrocatalyst due to their higher affinity for nitrogen, as observed from the nitrogenase enzymes with Fe-Mo cofactors (Liu et al., 2020; Arif et al., 2023). The nitrogenase enzymes exhibit dominant HER, which makes them unfavorable for efficient ammonia production (Hinnemann and Nørskov, 2006). Several strategies were followed to design Mo-based electrocatalysts to achieve effective NRR performances (Guo et al., 2021). As an important design strategy, MoS_2_ was synthesized as an NRR electrocatalyst which exhibits a sufficient ammonia yield but poor FE due to dominant HER (Zhang et al., 2018a). Therefore, Mo-based electrocatalysts must be engineered to achieve a higher ammonia yield and effectively suppress HER. In this way, Yesudoss et al. (Yesudoss et al., 2021) synthesized the γ-Mo_2_N supported on h-BN sheets as an effective NRR electrocatalyst. γ-Mo_2_N/BN hybrids, with strong interactions and bridging bonds between Mo_2_N and BN, exhibit a superior NRR performance. Therefore, precise tuning of the electrocatalyst design can enhance the NRR activity with a high ammonia yield rate and FE.

Typically, Mo_2_C electrocatalyst exhibits a Pt-like behavior, metallic nature, and better stability (Huo et al., 2016; Su et al., 2019). Thereby, Mo_2_C nanodots embedded in carbon nanosheets (Mo_2_C/C) were analyzed as an electrocatalyst for NRR, which initially revealed better ammonia yield even under strong hydrogen spillover (Cheng et al., 2018). This is attributed to the higher density of active sites in the Mo_2_C/C electrocatalyst, which exhibited a low FE due to higher selectivity for HER. Henceforth, these electrocatalysts are engineered with Mo single-atom catalysts (MoSAs-Mo_2_C) (Ma et al., 2020), MoO_2_ quantum dots (Mo_2_C-MoO_2_) (Wan et al., 2022), and as Mo_2_C nanorods (Ren et al., 2019), which reveal significantly high NRR performances. Notably, MoS_2_/Mo_2_C heterostructures were designed recently, which exhibited a high NRR FE of about 42% due to the selectivity of the Mo atoms towards N_2_ adsorption (Ye et al., 2023). However, a low ammonia yield rate of nearly 1.41 μg h^-1^ cm^-2^ was observed. Besides, Mo_2_N was identified as an effective electrocatalyst due to its high conductivity and ample active sites for redox reactions (Hargreaves, 2013; Mtukula et al., 2017). Therefore, Mo_2_N was studied as an NRR electrocatalyst, which exhibited a high ammonia yield five times more than the prepared MoO_2_ electrocatalyst indicating the supreme activity of the N atoms in Mo lattice (Ren et al., 2018). Besides, Mo_2_N electrocatalyst exhibited significant selectivity towards ammonia indicating the reliability of the Mo_2_N electrocatalyst (Ren et al., 2018; Yesudoss et al., 2021). Even though, the Mo_2_C and Mo_2_N electrocatalysts exhibit considerable electrocatalytic NRR performances, the electrocatalysts actively performs HER resulting in decreased NRR efficiency. Therefore, the electrocatalysts must be engineered carefully to achieve higher NRR activity.

Consequently, tailoring the electrocatalysts through a heterostructure strategy can be adopted, which increases the electrocatalytic activity by synergizing the performances of two different structures (Su et al., 2019; Wan et al., 2022). Heterostructures provide structural and electronic modifications in the electrocatalysts by which tunable performances are achieved, which proliferate the electrocatalytic performances (Yuan et al., 2022). For instance, Chen et al. (Chen et al., 2022) modified the MoS_2_ electrocatalyst with *in situ* generated MoO_2_ and ZnO (MMZ) as heterostructures, revealing significantly better NRR performances. The MMZ electrocatalyst exhibited a five-fold increase in ammonia yield and FE compared to pristine MoS_2,_ which indicates the multiplied electrocatalytic activity of the MMZ heterostructures. Therefore, the heterostructure formation has been a promising strategy in tailoring the performances of the pristine moieties to achieve considerably higher NRR performances. Besides, Co_3_O_4_-Mo_2_N heterostructures have revealed a high electrocatalytic activity due to the interfacial electronic effects between the two structures (Wang et al., 2022). The differential electron density between Mo_2_N and Co_3_O_4_ phases has improved the electrocatalytic activity of the heterostructures. Several other reports have suggested improved electrocatalytic activity by choice of suitable nitride heterostructures as an efficient strategy for NRR electrocatalysts (Yesudoss et al., 2021; Chu et al., 2022). Hence, the Mo-based electrocatalysts can be tailored with appropriate elements to achieve significantly higher ammonia yield rate and efficiency.

The above studies have motivated the fabrication of the single-phase transition metal carbides and nitrides into a heterostructure as an effective NRR electrocatalyst. In the present work, we demonstrate the preparation of the Mo_2_C-Mo_2_N_0.92_ nanocomposites and the analysis of NRR performances of the prepared electrocatalysts. Mo_2_C-Mo_2_N_0.92_ nanocomposites reveal the heterostructure formation of Mo_2_C and Mo_2_N structures. The prepared Mo_2_C-Mo_2_N_0.92_ electrocatalyst achieves a high ammonia yield rate of about 9.6 μg h^-1^ cm^-2^ and FE of nearly 10.15%, respectively. The enhanced NRR performances can be ascribed due to the synergistic activity of the heterostructures of Mo_2_C and Mo_2_N_0.92_, respectively. Hence, this study suggests promising electrocatalytic performances of the heterostructured electrocatalyst by tuning the structural and electronic properties of the single moieties.

## 2 Experimental methods

### 2.1 Synthesis procedure of Mo_2_C-Mo_2_N

Typically, a stoichiometric ratio of the precursors, molybdenum chloride (MoCl_5_) and urea (CO(NH_2_)_2_) were mixed together with 10 ml ethanol and aged on a hot plate at 80°C for 2–3 h. The as-prepared samples were further pyrolyzed at 800°C for 3 h under N_2_ flow. Further, the samples were prepared by varying the urea-metal ratio to 2:1 and 3:1, termed Mo_2_C-Mo_2_N_0.92_ and Mo_2_N_0.92_-Mo_2_C, respectively.

### 2.2 Characterizations

The crystal structure and phase formation of the samples were examined by powder X-ray diffraction (XRD; Rint 1000, Rigaku, Japan) with Cu Kα radiation (λ = 1.5418 Å). X-ray photoelectron spectroscopy (XPS; K-ALPHA+, Thermo Scientific) was used to determine the oxidation states of the elements in the electrocatalyst. The morphology and microstructure of the electrocatalyst was investigated by scanning electron microscopy (SEM; JSM-7500F, JEOL, Japan) and transmission electron microscopy (TEM; JEM-2100F, JEOL, Japan), respectively.

The slurry prepared using PVDF, carbon black and prepared electrocatalysts in the ratio 1:1:8 was loaded on carbon cloth (1 × 1 cm^2^) and dried in vacuum oven for further electrochemical studies. The electrochemical NRR studies were carried out in a three-electrode cell configuration using the VMP3-Biologic multi-channel potentiostat under 0.1 M KOH electrolyte with and without N_2_ purging. Graphite rod and Hg/HgO were used as the counter and reference electrodes, respectively. The Hg/HgO potential (E_(Hg/HgO)_) was converted into reversible hydrogen electrode (RHE) potential (E_RHE_) using Equation 1.

$$E_{RHE}=E_{\left(Hg/HgO\right)}+0.118\;V+0.0591\;x\;pH$$ (1)

### 2.3 Quantitative evaluation of NRR

The ammonia content in the electrolyte after the chronoamperometry (CA) tests for 2 h at various applied potentials were measured using the colorimetric method. 5 ml of the collected electrolyte after CA tests was sampled with colouring agents and catalysts using the standard ammonia test kits. Then the intensity of the coloured solution was measured using the UV-vis spectrophotometer. The intensity of the respective UV curves at 680 nm were compared to the standard calibration graphs of ammonia to estimate the concentration of ammonia present in the solution. The calibration graphs were derived from UV curves prepared by various known concentration of standard NH_4_Cl salt. The linear curves from the UV plots yield the calibration graphs for ammonia. Hence, the concentration of ammonia can be estimated from the UV curves for samples after CA tests and calibration graphs (Liu et al., 2020).

The ammonia yield rate and Faradaic efficiency (FE) of the prepared electrocatalysts can be estimated using Equation 2 and Equation 3, respectively (Liu et al., 2020).

$$Yield\;rate\;\left(\mu g\;h^{-1}{cm}^{-2}\right)=\frac{\left[{NH}_{3}\right]\;\left(\frac{\mu g}{ml}\right)\times V\left(ml\right)}{t\;\left(h\right)\times A\;\left({cm}^{-2}\right)}$$ (2)

$$FE\;\left(\%\right)=\frac{3\times Yield\;\left(\mu g\;h^{-1}{cm}^{-2}\right)\times F\;\left(C\;{mol}^{-1}\right)}{17.03\;\left(\frac{g}{mol}\right)\times j\;\left(mA\;{cm}^{-2}\right)}$$ (3)

where, [NH_3_] represent the concentration (μg/ml) of the ammonia present in the corresponding electrolyte, V is the volume of the electrolyte (ml), t is the time of electrolysis (hours), A is the area of the electrode (cm^-2^), F is the Faradaic constant (96485 C mol^-1^), and j is the current density at CA tests (mA cm^-2^), respectively.

## 3 Results and discussion

### 3.1 Structural and morphological analysis

**FIGURE 1:**
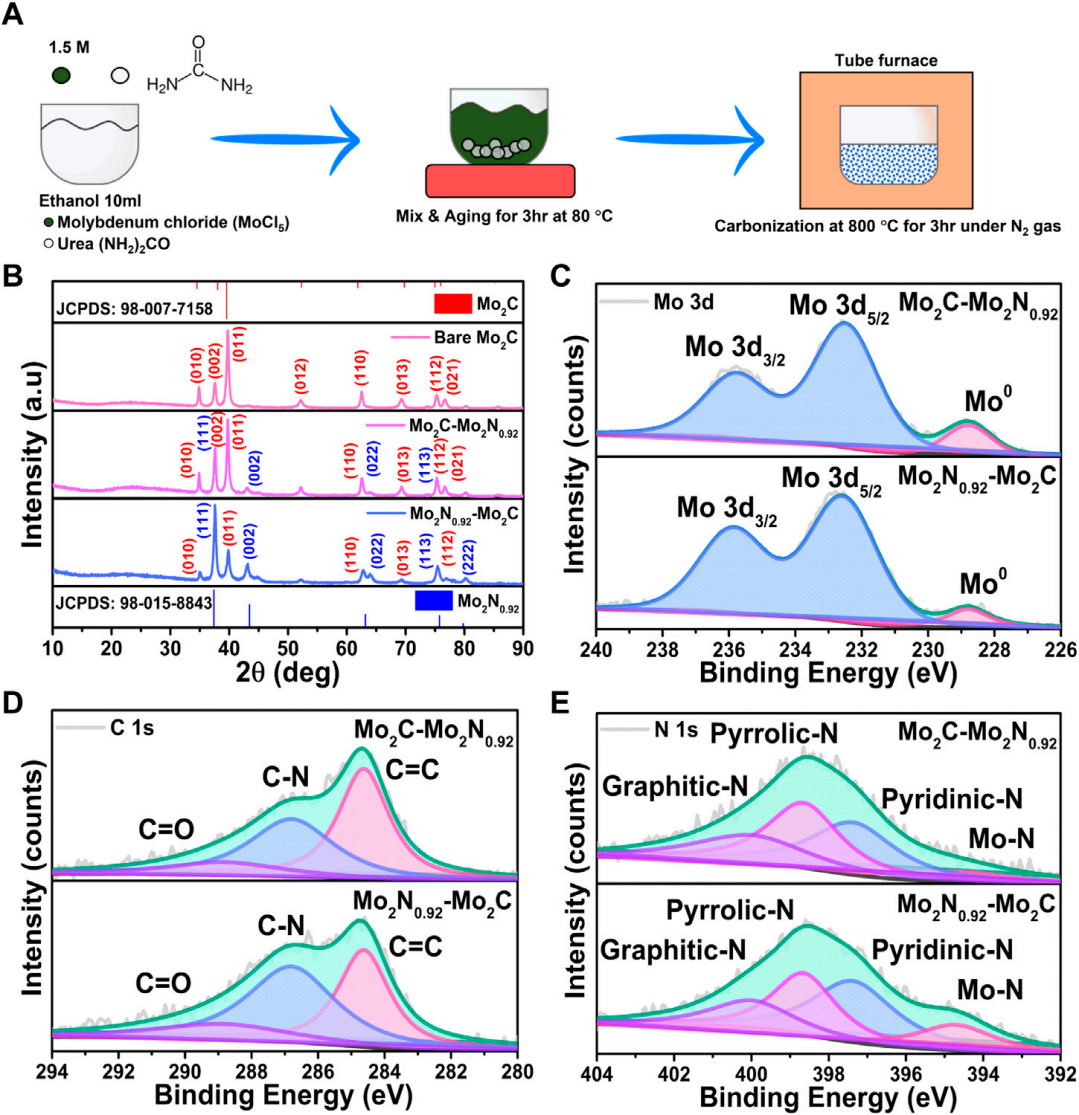
**(A)** Schematic representation of the synthesis procedure for Mo_2_C-Mo_2_N nanocomposites. **(B)** XRD patterns for the prepared Mo_2_C-Mo_2_N_0.92_, Mo_2_N_0.92_-Mo_2_C and bare Mo_2_C samples, respectively. XPS spectra of **(C)** Mo 3d, **(D)** C 1s, and **(E)** N 1s for the prepared Mo_2_C-Mo_2_N_0.92_ and Mo_2_N_0.92_-Mo_2_C samples.

Mo_2_C-Mo_2_N_0.92_ is synthesized by the simple urea glass route (Figure 1A). Initially, the MoCl_5_ precursors are mixed with alcohols which primarily forms Mo-ethoxide (Mo(OC_2_H_5_)_5_) and the Cl¯ ions are released as HCl (Giordano et al., 2008). Further, mixing urea (CO(NH_2_)_2_) with the above solution replaces the ethoxide groups in Mo(OC_2_H_5_)_5_ due to the high solubility of urea under the presence of metal ions (Equation 4). Urea is used as both C and N precursors for the formation of Mo_2_C and Mo_2_N, respectively. The C=O bond cleavage in urea replaces the ethoxide molecule in Mo(OC_2_H_5_)_5,_ and the urea moiety coordinates with the Mo^5+^ atom forming metal-urea complexes, Mo-(OCN_2_H_4_)_5_ (Equation 5). (Sardar et al., 2005) After heat treatment, the metal-urea complex is reduced into the desired Mo_2_N-Mo_2_C nanocomposites (Equation 6). By varying the urea-to-metal ratio (R), dominant phases of Mo_2_N and Mo_2_C can be achieved, respectively. Thereby, a lower urea content in the urea-metal ratio results in the dominant phases of Mo_2_C, and high urea content favors Mo_2_N formation respectively.

The proposed reaction mechanisms are elucidated as follows.

$${MoCl}_{5}+C_{2}H_{5}OH\;\to \;Mo{\left({OC}_{2}H_{5}\right)}_{5}+HCl$$ (4)

$$Mo{\left({OC}_{2}H_{5}\right)}_{5}+CO{\left({NH}_{2}\right)}_{2}\;\to \;Mo-{\left({OCN}_{2}H_{4}\right)}_{5}$$ (5)

$${Mo}^{5+}+R\;{OCN}_{2}H_{4}\;\to \;{Mo}_{2}C-{Mo}_{2}N+{NH}_{3}+HCN+{CO}_{2}$$ (6)

The structural analysis of the prepared samples was evaluated by X-ray diffraction (XRD) analysis. XRD patterns obtained for Mo_2_C-Mo_2_N_0.92_ and Mo_2_N_0.92_-Mo_2_C display crystalline peaks for the prepared samples (Figure 1B). The obtained peaks are well-matched to the respective phases of Mo_2_C (JCPDS: 98–007–7158) and Mo_2_N_0.92_ (JCPDS: 98–015–8843). Similarly, XRD patterns for the bare Mo_2_C samples reveal the respective well-matched phases. The simultaneous existence of the XRD patterns of the two phases without any peak shift or peak broadening can ascribe to the heterostructure formation. XRD patterns for Mo_2_C-Mo_2_N_0.92_ prepared with 2:1 ratio reveals the dominant phases of Mo_2_C with relatively smaller presence of Mo_2_N_0.92_ phases. Similarly, XRD patterns for Mo_2_N_0.92_-Mo_2_C prepared with 3:1 urea to metal ratio displays the presence of dominant Mo_2_N_0.92_ phases with feeble Mo_2_C phases, respectively. Thereby, a low urea content favors Mo_2_C formation and a high urea content forms Mo_2_N_0.92_, respectively. Hence, Mo_2_N_0.92_ and Mo_2_C phases exist together in a single system exhibiting differences in relative intensity of the XRD patterns without any peak shift or peak broadening. The relative intensity of the XRD patterns for the samples prepared at different urea to metal ratio explain the dominant phases of the Mo_2_C and Mo_2_N_0.92_ moieties in the prepared electrocatalysts. Thus, the urea-metal ratio is the phase-determining factor for Mo_2_N and Mo_2_C.

The chemical states of the prepared Mo_2_C-Mo_2_N_0.92_ and Mo_2_N_0.92_-Mo_2_C samples were evaluated by the X-ray photoelectron spectroscopy (XPS) analysis. The Mo 3d spectra in Figure 1C can be deconvoluted into two peaks at 232.8 eV and 236 eV for Mo 3d_5/2_ and Mo 3d_3/2_, respectively. The small peak arising at the lower binding energy around 228.8 eV corresponds to the metallic Mo species for both Mo_2_C-Mo_2_N_0.92_ and Mo_2_N_0.92_-Mo_2_C samples. The C 1s spectra in Figure 1D display three deconvoluted peaks at 284.6 eV, 286.8 eV, and 289 eV corresponding to the sp-carbon (C=C) bonds (Yue et al., 2021), N doped carbon (C-N), and feeble C=O signals due to incomplete decomposition of urea (CO(NH_2_)_2_), respectively. The deconvoluted N 1s spectra in Figure 1E display major peaks at 397.4 eV, 398.7 eV, and 400 eV, corresponding to pyridinic-N, pyrrolic-N, and graphitic-N, respectively. Besides, the feeble peak around 394.8 eV suggests the presence of Mo-N bonds in the prepared samples (Zhang et al., 2022). Evidently, the Mo_2_N_0.95_-Mo_2_C samples reveal relatively higher intensity for Mo-N bonds compared to Mo_2_C-Mo_2_N_0.92_, which validates the dominant presence of Mo_2_N_0.92_ in the prepared sample.

**FIGURE 2:**
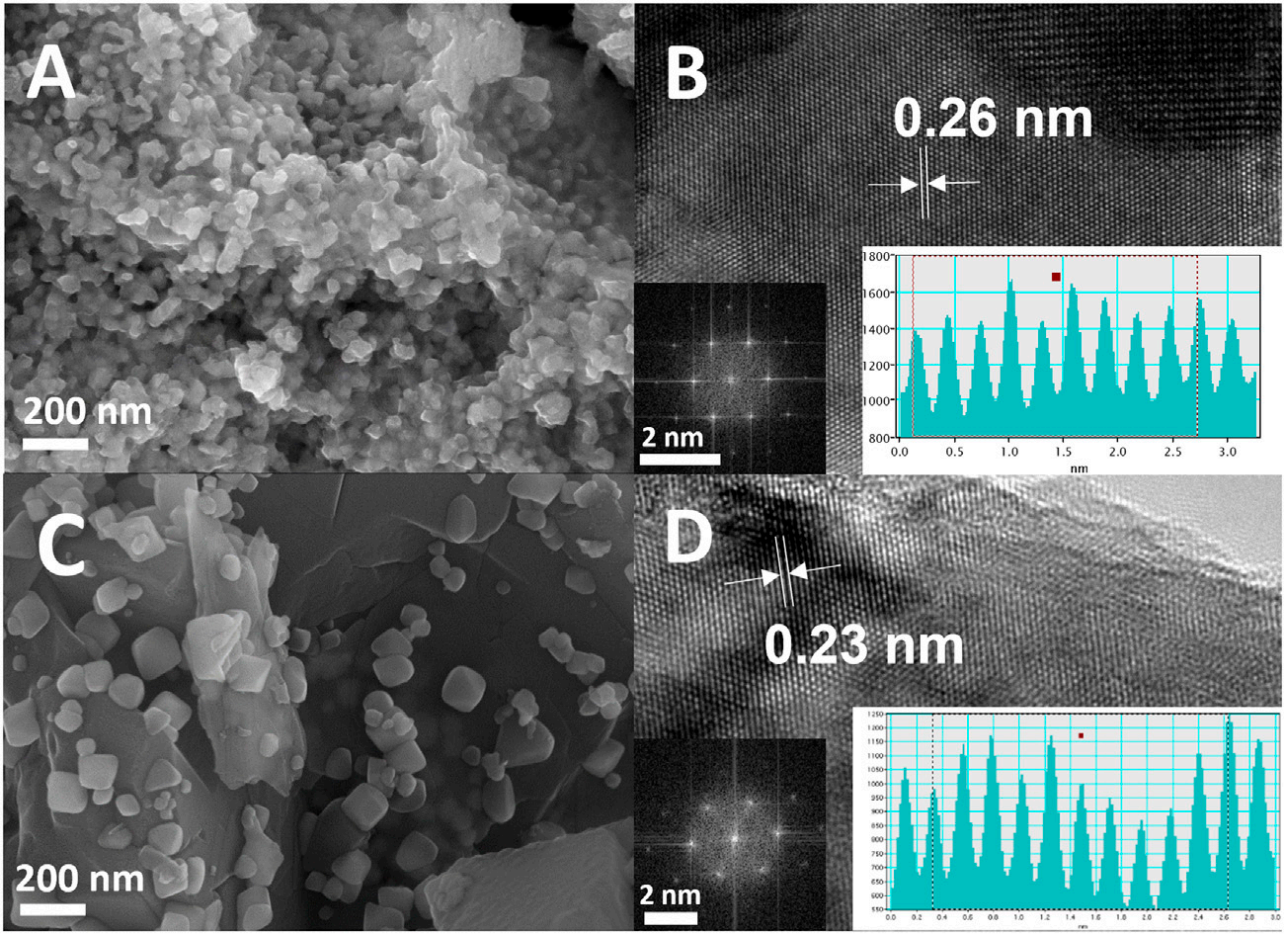
**(A)** SEM image, **(B)** HRTEM image (inset: FFT analysis and intensity-line profile) for Mo_2_C-Mo_2_N_0.92_ sample, **(C)** SEM image, **(D)** HRTEM image (inset: FFT analysis and intensity-line profile) for Mo_2_N_0.92_-Mo_2_C sample.

The morphological and microstructure studies were observed using scanning electron microscopy (SEM) and high-resolution transmission electron microscopy (HRTEM) analyses. SEM images for the prepared Mo_2_C-Mo_2_N_0.92_ samples reveal the uniform distributions of the nanocomposites anchored to a substrate. SEM images of Mo_2_C-Mo_2_N_0.92_ sample reveal the average sizes of the nanocomposites around ∼50 nm (Figure 2A). HRTEM analysis reveals the lattice spacing of about 0.26 nm for Mo_2_C-Mo_2_N_0.92_ nanocomposites corresponding to (010) lattice planes of Mo_2_C (Figure 2B). The inset of Figure 2B shows the FFT patterns for the Mo_2_C-Mo_2_N_0.92_ sample, which reveal the crystalline phases of Mo_2_C. Similarly, the SEM images in Figure 2C reveal the average sizes of the nanocomposites around ∼110 nm for Mo_2_N_0.92_-Mo_2_C. The HRTEM analysis for Mo_2_N_0.92_-Mo_2_C sample reveals the lattice spacing of about 0.23 nm corresponding to (101) lattice planes of Mo_2_C (Figure 2D). Furthermore, the intensity-line profiles for Mo_2_C-Mo_2_N_0.92_ and Mo_2_N_0.92_-Mo_2_C samples, as shown in the inset of Figures 2B, D, display an average interplanar distance of nearly 0.26 nm for Mo_2_C-Mo_2_N_0.92_ and 0.23 nm for Mo_2_N_0.92_-Mo_2_C, respectively.

### 3.2 Electrochemical NRR performance

**FIGURE 3:**
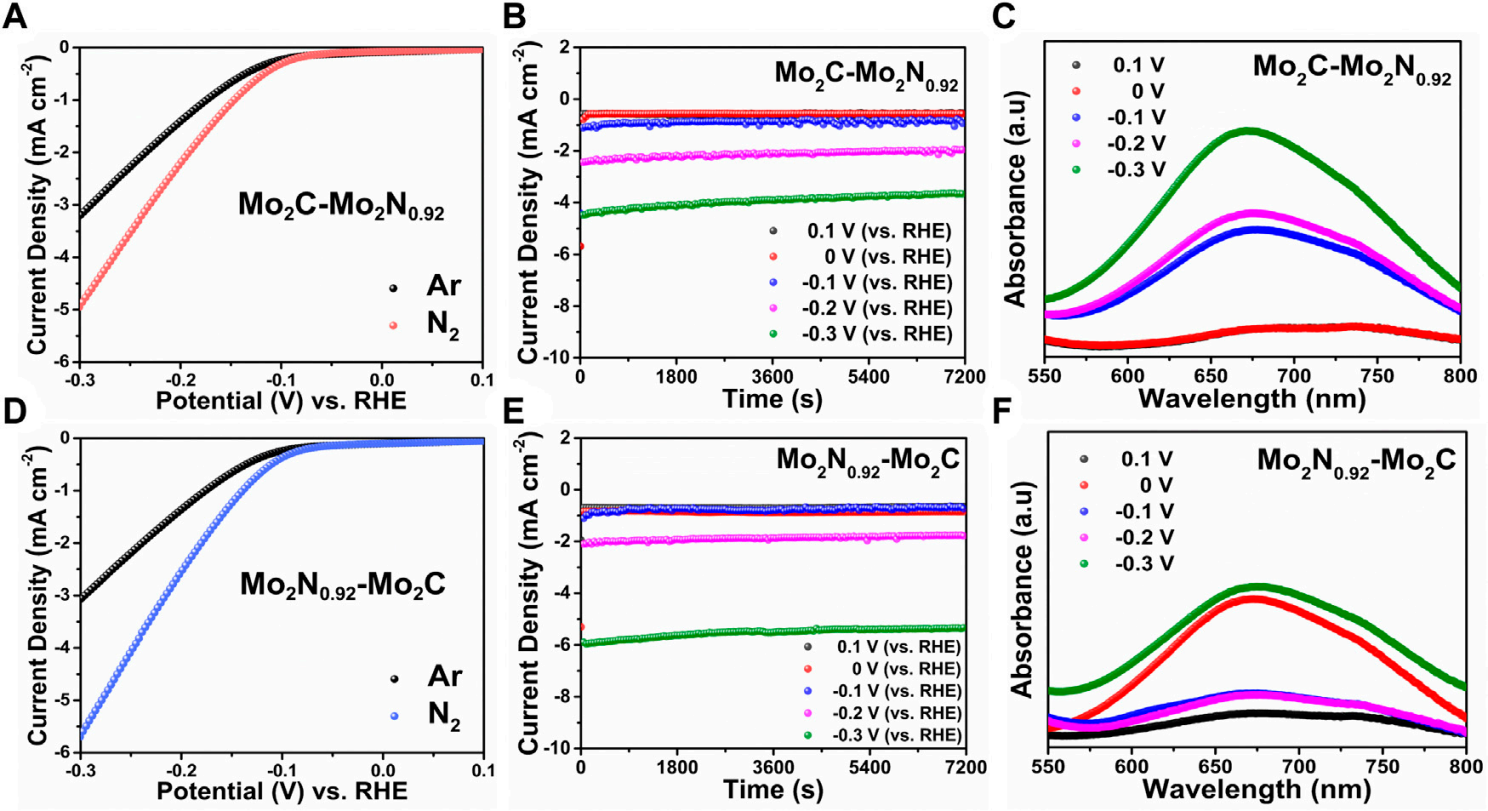
**(A)** LSV curves, **(B)** CA tests at various applied potentials, **(C)** UV-vis plots for corresponding CA test samples for Mo_2_C-Mo_2_N_0.92_ electrocatalyst. **(D)** LSV curves, **(E)** CA tests at various applied potentials, and **(F)** UV-vis plots for corresponding CA test samples for Mo_2_N_0.92_-Mo_2_C electrocatalyst.

Electrochemical NRR performances of the prepared electrocatalysts were performed under constant N_2_ purging. Linear sweep voltammetry (LSV) performed under Ar- and N_2_-saturated electrolyte reveals a lower onset with higher current density under N_2_ saturation for Mo_2_C-Mo_2_N_0.92_ electrocatalyst due to the occurrence of NRR (Figure 3A). NRR performances were quantified by the chronoamperometric (CA) measurements performed for 2 h under N_2_-saturation at various applied potentials. The electrolytes were sampled for ammonia quantification by UV-colorimetric analysis using a commercial ammonia test kit. The ammonia calibration graphs were derived using known ammonia concentrations with a UV-vis spectrometer (Supplementary Figure S1). UV-vis spectra for the sampled electrolytes under various potentials were observed to display varied intensities indicating the concentration of ammonia. The ammonia concentration was calculated from the calibration graphs, and the ammonia yield rate and FE from Equation 2 and Equation 3, respectively. Figures 3B, C represent the CA curves and UV plots for Mo_2_C-Mo_2_N_0.92_ electrocatalyst at various applied potentials, respectively. Similarly, the LSV curves for Mo_2_N_0.92_-Mo_2_C electrocatalyst reveal a higher current density under N_2_-saturation, indicating NRR (Figure 3D). Figures 3E, F represents the CA plots and corresponding UV curves for the prepared Mo_2_N_0.92_-Mo_2_C electrocatalyst for ammonia quantification.

**FIGURE 4:**
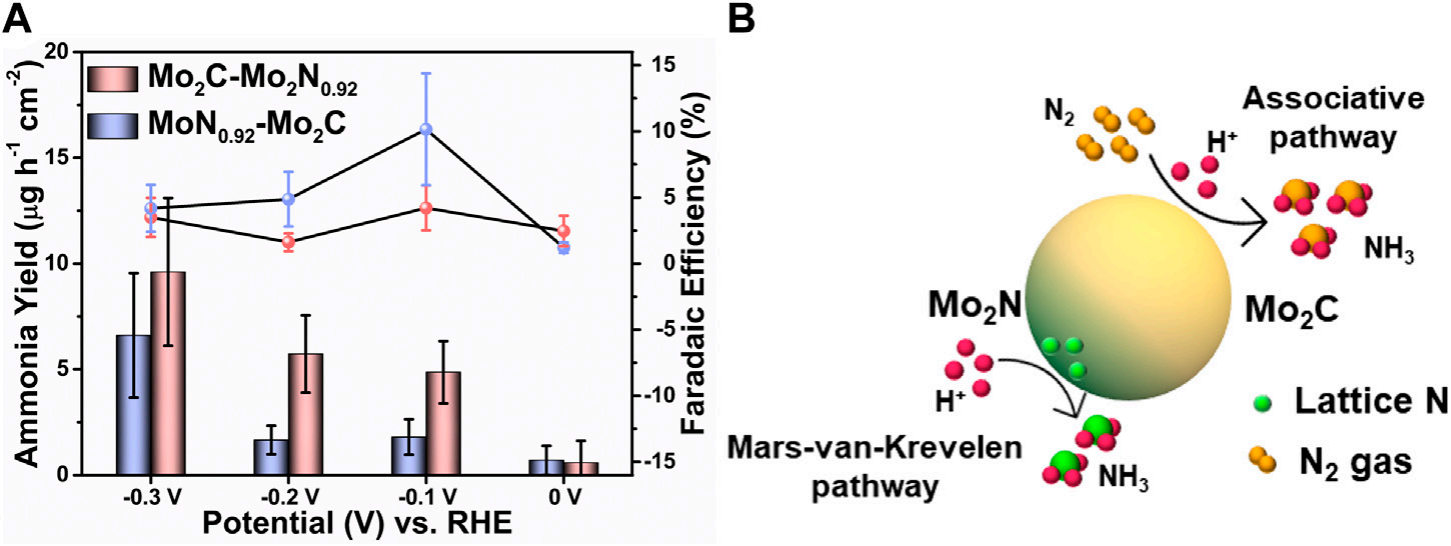
**(A)** Comparison of the ammonia yield and FE of Mo_2_C-Mo_2_N_0.92_ and Mo_2_N_0.92_-Mo_2_C electrocatalysts for various applied potentials. (Error bars indicate the range of values after three independent experiments) **(B)** Schematic illustration of NRR mechanism over Mo_2_C-Mo_2_N electrocatalyst.

Figure 4A represents ammonia yield rates and FE of Mo_2_C-Mo_2_N_0.92_ and Mo_2_N_0.92_-Mo_2_C electrocatalysts for various applied potentials, respectively. A maximum ammonia yield of about 9.6 μg h^-1^ cm^-2^ and 6.61 μg h^-1^ cm^-2^ at −0.3 V vs. RHE was observed for Mo_2_C-Mo_2_N_0.92_ and Mo_2_N_0.92_-Mo_2_C electrocatalyst, respectively. Consequently, a maximum FE of about 10.15% and 4.21% at −0.1 V vs. RHE was observed for Mo_2_C-Mo_2_N_0.92_ and Mo_2_N_0.92_-Mo_2_C electrocatalyst, respectively. A comparatively high current density was observed under N_2_ flow but a lower FE for ammonia of about 4.21% was observed at −0.1 V vs. RHE for Mo_2_N_0.92_-Mo_2_C electrocatalyst. HER is kinetically favorable due to the comparatively low potential required for H_2_ evolution than to dissociate the stable triple bonds of N_2_. Thereby, the low FE for ammonia can be attributed to the undesired HER with increasing negative potentials. Moreover, the particle sizes of Mo_2_C-Mo_2_N_0.92_ and Mo_2_N_0.92_-Mo_2_C from SEM images were observed to be 50 nm and 110 nm, respectively. It is suggested that a smaller size of the particles enables higher surface area and high exposure of active sites to drive the required electrochemical reactions. Consequently, Mo_2_C-Mo_2_N_0.92_ electrocatalysts with a particle size of about 50 nm exhibits a higher NRR performance compared to Mo_2_N_0.92_-Mo_2_C electrocatalysts with a higher particle size of about 110 nm. Therefore, particle sizes play an important role in determining the effective NRR performances for Mo_2_C-Mo_2_N_0.92_ electrocatalysts.

Besides, a comparatively lower ammonia yield and FE was observed for Mo_2_N_0.92_-Mo_2_C electrocatalyst, which can be due to the less activity of Mo_2_N_0.92_ phases for N_2_ reduction. The NRR performances for bare Mo_2_C and bare Ni foam were analyzed, which reveal a maximum ammonia yield of about 4.53 μg h^-1^ cm^-2^ and 0.07 μg h^-1^ cm^-2^ and FE of about 0.14% and 0.03%, respectively (Supplementary Figure S2). This poor NRR performance for bare Mo_2_C and bare Ni foam substrate indicates the synergistic activity of the Mo_2_C-Mo_2_N_0.92_ electrocatalyst. Figure 4B illustrates the NRR mechanism in the prepared Mo_2_C-Mo_2_N_0.92_ heterostructures. The Mo_2_C phases in the Mo_2_C-Mo_2_N_0.92_ electrocatalyst generate ammonia by the electrochemical associative pathway by protonating the external N_2_ gas and the Mo_2_N phases following the MvK pathway by protonating the lattice-N to ammonia.

Mo_2_C-Mo_2_N_0.92_ electrocatalyst has revealed considerably higher NRR performances due to the combined activity of the Mo_2_C and Mo_2_N_0.92_ phases. N_2_ adsorption and activation over an electrocatalyst surface occur at the exposed active sites. Previous studies have observed a strong N_2_ adsorption behavior for Mo_2_C, which reveals a promising NRR electrocatalyst (Wan et al., 2022). Especially, the electron-rich Mo atoms with moderate H adsorption energy and high N_2_ affinity are the main electrocatalytic active sites for NRR (Ma et al., 2020; Liu et al., 2021; Zhang et al., 2018a).

NRR takes place by the ‘acceptance-donation’ mechanism at the Mo active sites is explained as follows. The lone pair of electrons from the σ-orbitals of N_2_ are donated into the empty d-orbitals of Mo, resembling acceptance, followed by the back-donation of the d-orbital electrons into the π* orbitals of N_2_, resembling donation (Fan et al., 2022; Wan et al., 2022). This acceptance-donation mechanism over Mo active centers results in the activation of the highly stable triple bonds of N_2_ and realizes an increase in bond length ((Mo)-N=N•), as shown in Equation 7. The chemisorbed N_2_ on Mo active sites is further protonated by six proton-coupled electron transfer steps (PCET) to form ammonia (Equation 8) by associative mechanism (Cheng et al., 2018; Ren et al., 2019).

$$Activation\;step:N_{2}+\left(Mo\right)\;\to \;\left(Mo\right)-N=N•$$ (7)

$$Overall\;reaction:\left(Mo\right)-N=N•+6\;H_{2}O+6\;e\overset{–}{}\to \;2\;{NH}_{3}+6\;OH^{–}$$ (8)

The associative mechanism for ammonia production over the Mo electrocatalytic active sites can be suggested as follows. Further, the activated N_2_ can follow either an alternating protonating pathway or a distal protonating pathway.

$$\left(Mo\right)-N=N•+H_{2}O+e^{–}\bar{}\to \;\left(Mo\right)-N=NH+OH^{–}\bar{}$$ (9)

Distal associative pathway

$$\left(Mo\right)-N=NH+H_{2}O+e\overset{–}{}\to \;\left(Mo\right)-N-{NH}_{2}+OH^{–}$$ (10)

$$\left(Mo\right)–N–{NH}_{2}+H_{2}O+e\overset{–}{}\to \;\left(Mo\right)–N–{NH}_{3}+OH^{–}\to \;\left(Mo\right)–N+{NH}_{3}\;↑$$ (11)

$$\left(Mo\right)-N+H_{2}O+e\;\overset{–}{}\to \;\left(Mo\right)-NH+OH^{–}$$ (12)

$$\left(Mo\right)-NH+H_{2}O+e\;\overset{–}{}\to \;\left(Mo\right)-{NH}_{2}+OH^{–}$$ (13)

$$\left(Mo\right)-{NH}_{2}+H_{2}O+e\;\overset{–}{}\to \;\left(Mo\right)-{NH}_{3}+OH^{–}\to \;\left(Mo\right)+{NH}_{3}\;↑$$ (14)

Alternating associative pathway

$$\left(Mo\right)-N=NH+H_{2}O+e\overset{–}{}\to \;\left(Mo\right)-NH-NH+OH^{–}$$ (15)

$$\left(Mo\right)-NH=NH+H_{2}O+e\overset{–}{}\to \;\left(Mo\right)-NH-{NH}_{2}+OH^{–}$$ (16)

$$\left(Mo\right)-NH={NH}_{2}+H_{2}O+e\overset{–}{}\to \;\left(Mo\right)-{NH}_{2}-{NH}_{2}+OH^{–}$$ (17)

$$\left(Mo\right)-{NH}_{2}-{NH}_{2}+H_{2}O+e\overset{–}{}\to \;\left(Mo\right)-{NH}_{2}-{NH}_{3}+OH^{–}\to \;\left(Mo\right)-{NH}_{2}+{NH}_{3}\;↑$$ (18)

$$\left(Mo\right)-{NH}_{2}+H_{2}O+H_{2}O+e\overset{–}{}\to \;\left(Mo\right)-{NH}_{3}\;\to \;\left(Mo\right)+{NH}_{3}\;↑$$ (19)

In the distal associative pathway, the protonation occurs from the farther side of the activated N atom (Equations 9-11). The protonation of the N atom bonded to the active site initiates only after complete protonation and ammonia evolution of the farthest N atom (Equations 12–14). In the alternating pathway, the protonation occurs alternatively on both N atoms and ammonia is generated at the final successive steps (Equations 15–19). Typically, the charge transfer reaction takes place *via* the charge accumulation and depletion stages in both Mo and N atoms attributing to better NRR performances (Wan et al., 2022).

Besides, Mo_2_N_0.92_ is suggested to generate ammonia by decomposition of the Mo_2_N lattice rather than by catalytic mechanism (Zhang et al., 2018b; Hu et al., 2019). Thereby, the Mo_2_N_0.92_ entity present in the Mo_2_C-Mo_2_N_0.92_ electrocatalyst generates ammonia by the Mars-van-Krevelen (MvK) mechanism. By the MvK mechanism, the lattice N atoms in metal nitride electrocatalysts are protonated rather than the conversion of gaseous N_2_ to form ammonia (Equations 20–22) (Yang et al., 2019). The detailed MvK mechanism over Mo_2_N phases is elucidated as follows.

$${Mo}_{2}N+H_{2}O+e\overset{–}{}\to \;{Mo}_{2}\left(NH\right)+OH^{–}$$ (20)

$${Mo}_{2}\left(NH\right)+H_{2}O+e\overset{–}{}\to \;{Mo}_{2}\left({NH}_{2}\right)+OH^{–}$$ (21)

$${Mo}_{2}\left({NH}_{2}\right)+H_{2}O+e\overset{–}{}\to \;{Mo}_{2}\left({NH}_{3}\right)+OH^{–}\to \;{Mo}_{2}*+{NH}_{3}\;↑$$ (22)

$${Mo}_{2}*+N_{2}\;\to \;{Mo}_{2}\left(N\equiv N\right)\;\left(Surface\;replenishment\right)$$ (23)

$${Mo}_{2}\left(N\equiv N\right)+H_{2}O+e\overset{–}{}\to \;{Mo}_{2}\left(N\equiv {NH}_{2}\right)+OH^{–}$$ (24)

$${Mo}_{2}\left(N=NH\right)+H_{2}O+e\overset{–}{}\to \;{Mo}_{2}\left(N-{NH}_{2}\right)+OH^{–}$$ (25)

$${Mo}_{2}\left(N–{NH}_{2}\right)+H_{2}O+e\overset{–}{}\to \;{Mo}_{2}\left(N–{NH}_{3}\right)+OH^{–}\bar{}\to {Mo}_{2}\left(N\right)+{NH}_{3}\;↑$$ (26)

Where Mo_2_* represents the nitrogen vacancy formed due to the MvK mechanism. The surface replenishment of the nitrogen vacancies from N_2_ occurs, which generates ammonia (Equation 23) (Abghoui et al., 2016). In cases due to low N_2_ solubility in aqueous electrolytes, the surface nitrogen vacancies are replaced by the N atoms from the bulk resulting in the degradation of the electrocatalyst over time. Thereby, the surface replenished N is then protonated to release ammonia (Equations 24–26). Besides, the MvK mechanism demands high energy for NRR, which facilitates HER at higher potentials (Ren et al., 2018). Nitrogen vacancies affect the neighboring N atoms in the lattice resulting in poor electrocatalytic performances (Ren et al., 2018). As a result, an increased ammonia yield due to the heterostructure formation can be ascribed to the combined contribution from electrochemical N_2_ reduction and the MvK mechanism. The NRR performances in the prepared Mo_2_C-Mo_2_N_0.92_ electrocatalysts reveal the combined activity of the Mo_2_C and Mo_2_N_0.92_ phases. Hence, the combined electrochemical associative mechanism for N_2_ reduction *via* Mo_2_C and the MvK pathway by lattice-N protonation *via* Mo_2_N_0.92_ resulted in a high ammonia yield compared to the pristine counterparts.

### 3.3 Electrochemical characterization

Electrochemical impedance spectroscopy (EIS) analysis was undergone to elucidate the impedance characteristics of the prepared electrocatalysts. The Nyquist plots for real part (Z′) and imaginary part (Z”) of impedance elucidate the charge transfer and diffusion characteristics of the prepared electrocatalysts. The initial intercept on Z’ axis at the high frequency region denotes the solution resistance (R_s_) in the electrode and electrolyte interface. Besides, the diameter of the semi-circle in the mid-frequency region denotes the charge transfer resistance (R_ct_) for the redox reactions between the active sites of the electrode and the electrolytic ions. Additionally, the linear curve in the low frequency region ascribes to the diffusion characteristics of the prepared electrocatalysts.

**FIGURE 5:**
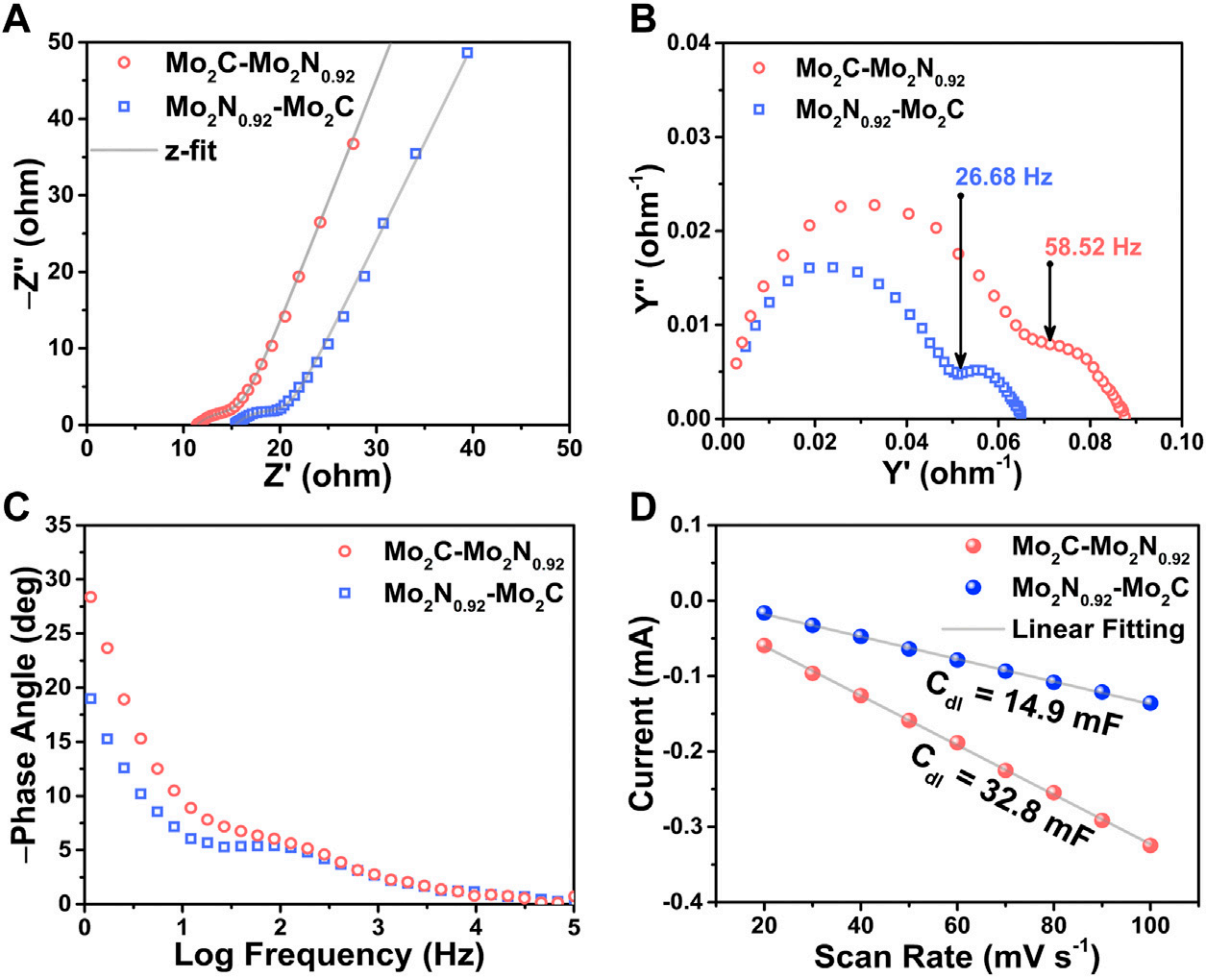
**(A)** Nyquist impedance plots, **(B)** Nyquist admittance plots, and **(C)** Bode plots for the prepared Mo_2_C-Mo_2_N_0.92_ and Mo_2_N_0.92_-Mo_2_C electrocatalysts, respectively. **(D)** Current vs. scan rate plots derived from CV curves performed at various scan rates.

From the Nyquist plots in Figure 5A, Mo_2_C-Mo_2_N_0.92_ electrocatalyst exhibits a low R_s_ value of about 11 Ω compared to Mo_2_N_0.92_-Mo_2_C (15 Ω) electrocatalyst. Besides, the Mo_2_C-Mo_2_N_0.92_ electrocatalyst reveals R_ct_ value of about 4.5 Ω which is very less compared to 6.5 Ω for Mo_2_N_0.92_-Mo_2_C electrocatalyst. Thereby, the Mo_2_C-Mo_2_N_0.92_ electrocatalyst is observed to exhibit faster electrocatalytic redox reactions superior to Mo_2_N_0.92_-Mo_2_C electrocatalyst. The superior electrocatalytic activity of the dominant Mo_2_C phases in Mo_2_C-Mo_2_N_0.92_ electrocatalyst explains the reduced resistance for redox reactions. Additionally, a higher angle for the linear curve with respect to *x*-axis was observed for Mo_2_C-Mo_2_N_0.92_ in the low frequency region compared to Mo_2_N_0.92_-Mo_2_C which indicate the superior diffusion characteristics of the Mo_2_C-Mo_2_N_0.92_ electrocatalyst.

The Nyquist admittance plots explain the conductivity of the prepared electrocatalysts as a function of frequency. The admittance plot is characterized by the knee frequency which elucidate the corresponding charge transfer resistances of the prepared electrocatalysts. From Figure 5B, the Mo_2_C-Mo_2_N_0.92_ electrocatalyst displays a higher knee frequency at 58.52 Hz compared to Mo_2_N_0.92_-Mo_2_C (26.68 Hz). The higher knee frequency substantiates the lower charge transfer resistance for Mo_2_C-Mo_2_N_0.92_ electrocatalyst which agrees with the Nyquist plots. The Bode phase plot in Figure 5C for the prepared electrocatalysts were obtained from the phase angle vs. frequency plots to elucidate the frequency response of the electrocatalyst. The phase angle denotes the phase difference between the current and voltage of the electrochemical cell. A phase angle of −90° denotes the ideal capacitor behavior elucidating a double-layer capacitance (C_dl_). The curves in the low frequency region of the Bode phase plot denote the double layer capacitance(C_dl_) of the electrocatalyst and the curves in high frequency region denote the capacitance in the redox reactions. Thereby, the Bode phase plots suggest a slight phase angle shift towards high frequencies for Mo_2_C-Mo_2_N_0.92_ electrocatalyst than Mo_2_N_0.92_-Mo_2_C indicating faster reaction kinetics. Hence, the dominant phases of Mo_2_C in Mo_2_C-Mo_2_N_0.92_ electrocatalyst drives a faster reaction kinetics than the Mo_2_N_0.92_ phases which validate the previous electrochemical studies.

Electrochemical active surface area (ECSA) of the prepared electrocatalysts were measured to estimate the available surface area for electrochemical reactions. ECSA was estimated by calculating the double layer capacitance (C_dl_) of the prepared electrocatalysts by performing CV tests for various scan rates in the non-faradaic region, as shown in Figure 5D (Supplementary Figure S3). The current response in the non-faradaic region is purely due to the charge/discharge of the double layer. Thereby, the C_dl_ value (mF) was estimated from the slope of the linear plot of scan rate vs. current density derived from the CV curves. Consequently, the ratio of the C_dl_ value and the specific capacitance (40 μF cm^-2^) yields ECSA value of the prepared electrocatalyst. As per the discussion, the electrocatalysts exhibit C_dl_ values of about 32.8 mF and 14.9 mF for Mo_2_C-Mo_2_N_0.92_ and Mo_2_N_0.92_-Mo_2_C, respectively. Consequently, the electrocatalysts exhibit a high ECSA value of about 820 cm^2^ and 372.5 cm^2^ for Mo_2_C-Mo_2_N_0.92_ and Mo_2_N_0.92_-Mo_2_C electrocatalyst, respectively. Thereby, a high ECSA value attributes to higher exposure of active sites to the electrolytic ions for redox reactions.

## 4 Conclusion

In summary, the prepared Mo_2_C-Mo_2_N_0.92_ electrocatalyst was synthesized by urea glass route under varying urea amounts followed by pyrolysis at 800°C under an N_2_ atmosphere. The structural analysis reveals the formation of well-matched phases for Mo_2_C-Mo_2_N_0.92_ samples respectively. The morphological and microstructure analysis reveals the formation of nanocomposites and well-defined lattice planes for Mo_2_C-Mo_2_N_0.92_. The chemical state analysis of the prepared samples reveal the presence of metallic Mo bonds in addition to Mo_2_N and Mo_2_C. The electron-rich Mo sites act as the active sites for electrocatalytic NRR activity. NRR performances reveal a maximum ammonia yield of around 9.6 μg h^-1^ cm^-2^ at −0.3 V vs. RHE and an FE of about 10.15% at −0.1 V vs. RHE for Mo_2_C-Mo_2_N_0.95_ electrocatalyst, respectively. The improved NRR performances were attributed to the synergistic activity of the Mo_2_C and Mo_2_N_0.92_ moieties, which is verified by the NRR activity of the single-phase Mo_2_C sample. Therefore, this study recommends the superior electrocatalytic activity of the tailored transition metal carbide and nitride electrocatalysts which could be further engineered to achieve efficient electrocatalysis.

## Data Availability

The raw data supporting the conclusions of this article will be made available by the authors, without undue reservation.
