# Supplementary material for: Promoting electrochemical ammonia synthesis by synergized performances of Mo2C-Mo2N heterostructure
Source: Front Chem. 2023 Feb 16;11:1122150. doi: 10.3389/fchem.2023.1122150 (PMC9980907; doi:10.3389/fchem.2023.1122150)
Supplement: Supplementary file 1 [file DataSheet1.docx]

Supplementary Material

Promoting Electrochemical Ammonia Synthesis by Synergized Performances of Mo_2_C-Mo_2_N Heterostructures via Efficient Electrochemical Nitrogen Reduction Reaction

Tae-Yong An^1,‡^, Subramani Surendran^1,‡^, Sebastian Cyril Jesudass^2^, Hyunjung Lee^1^, Dae Jun Moon^1^, Jung Kyu Kim^3,^* and Uk Sim^1,4,5,^*

^1^ Hydrogen Energy Technology Laboratory, Korea Institute of Energy Technology (KENTECH), 200 Hyeoksin-ro, Naju, Jeonnam 58330, Rep. of Korea.

^2^ Department of Material Science and Engineering, Chonnam National University, Gwangju 61186, Rep. of Korea.

^3^ School of Chemical Engineering, Sungkyunkwan University, 2066 Seobu-ro, Jangan-gu, Suwon, 16419, Rep. of Korea

^4^ Research Institute, NEEL Sciences, INC., 58326 Jeollanamdo, Rep. of Korea.

^5^ Center for Energy Storage System, Chonnam National University, Gwangju 61186, Rep. of Korea.

^‡^ These authors contributed equally.

*** Correspondence:** Corresponding Author: usim@kentech.ac.kr (Uk Sim)

Keywords: Ammonia Electrosynthesis; Electrocatalyst, Electrochemical Nitrogen reduction reaction

# Preparation of working electrode

A homogeneous slurry was prepared by mixing polyvinylidene difluoride (PVDF), carbon black and active material in a 1:1:8 ratio. The prepared slurry was brush-coated onto a carbon cloth substrate to prepare the working electrode and dried in an 80 °C oven for 12 hours.

# Determination of Ammonia

# 2.1 Calculation of NH_3_ yield rate and Faradaic efficiency

The NH_3_ yield rate corresponds to the yield of NH_3_ of the catalyst during the NRR test. It was calculated using the following equation:

*ʋ_NH3_ = C_NH3_.V/m_cat_.t* (S1)

Where C_NH3_ is the concentration of NH_3_ (unit: g L^-1^) in the electrolyte after NRR test, V is the volume of the electrolyte (unit: L); in this study, m_cat_ is the loading mass of the Au@Cu_2_Se (unit: g), t is the reaction time (unit: h).

FE means the percentage of the electric charge used for N_2_ reduction compared to the total electric charge used during the NRR test, which can be obtained by the following equation:

FE= CNH3.V.N.F/17.QFE= CNH3.V.N.F/17.Q (S2)

Where N is the number of electrons used to NH_3_ production, which equals 3, F refers to Faraday’s constant (96, 485 C mol^-1^), Q is the total electric charge during the NRR test (unit: C).

## Supplementary Figures


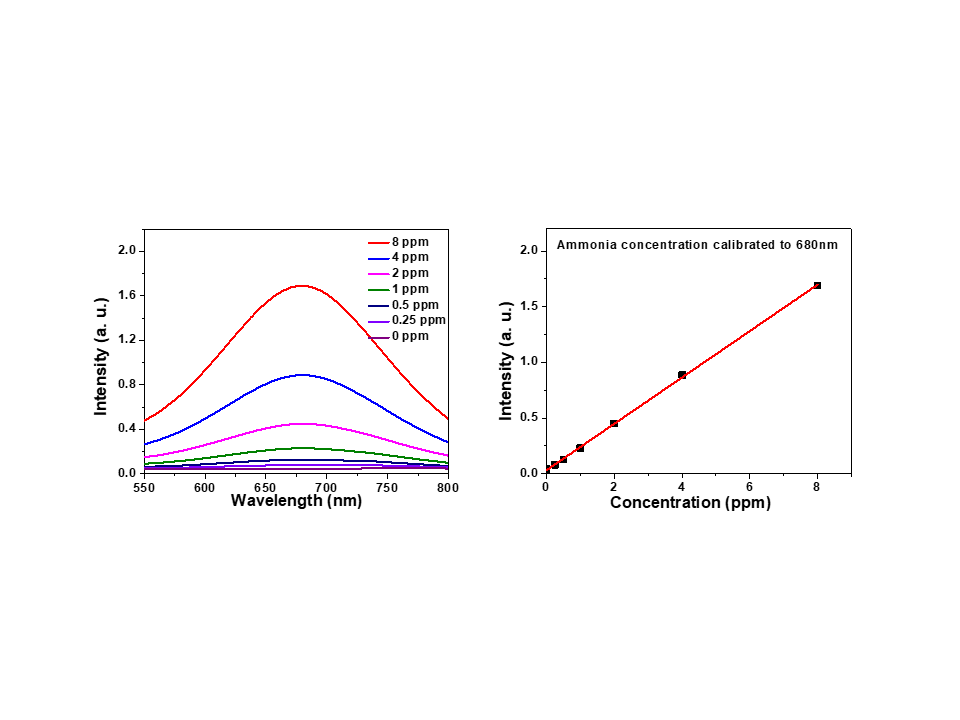


**Figure S1** a. UV plots for various known concentrations of ammonia using standard NH_4_Cl_­_ salt, b. Corresponding calibration plots taken at 680 nm.


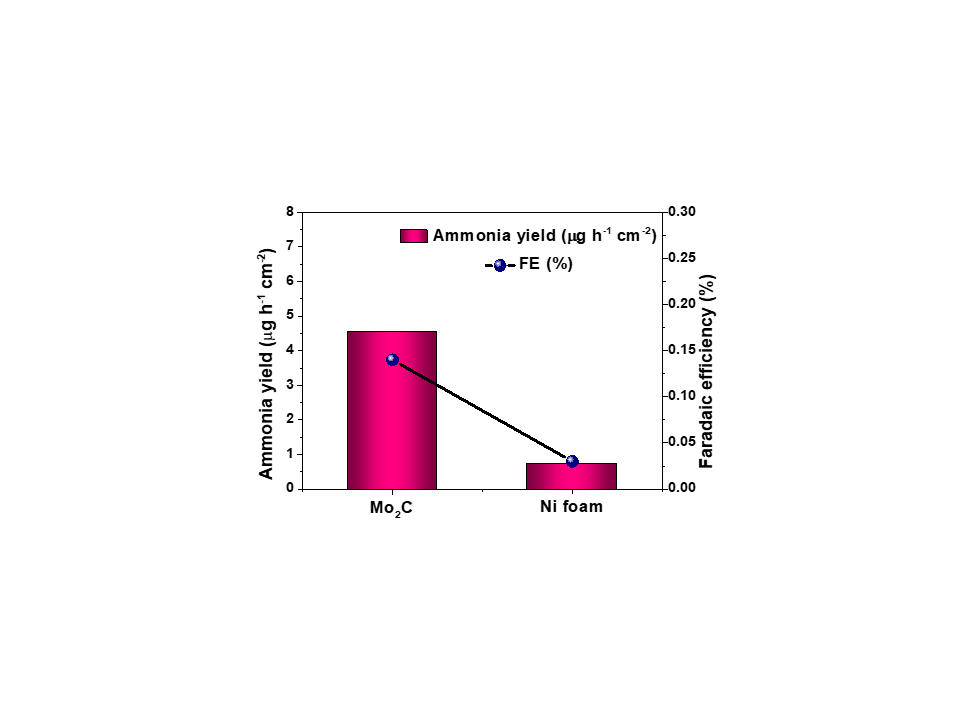


**Figure S2** Comparison of the ammonia yield and FE for Mo_2_C and Ni foam, respectively.


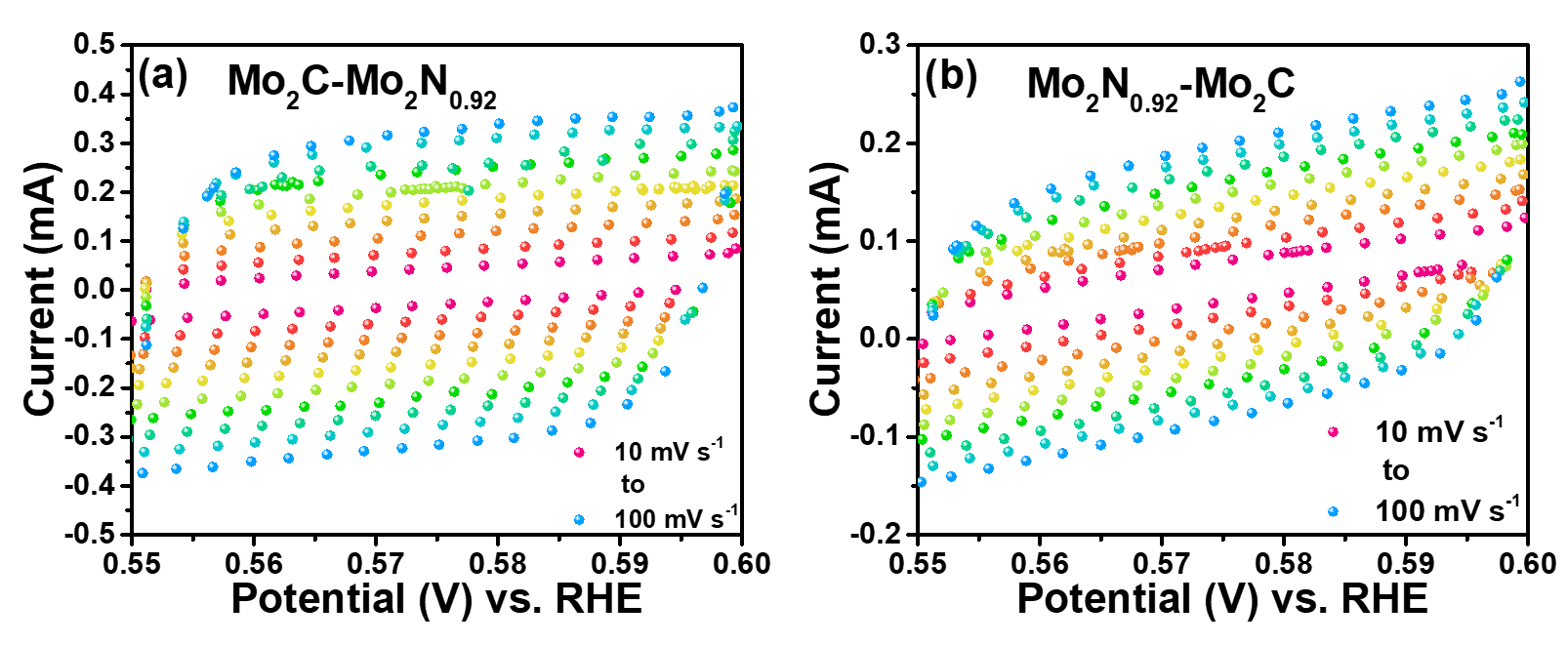


**Figure S3 (a,b) CV curves of prepared samples.**
